# Sex Effect on Presurgical Language Mapping in Patients With a Brain Tumor

**DOI:** 10.3389/fnins.2020.00004

**Published:** 2020-01-24

**Authors:** Shun Yao, Einat Liebenthal, Parikshit Juvekar, Adomas Bunevicius, Matthew Vera, Laura Rigolo, Alexandra J. Golby, Yanmei Tie

**Affiliations:** ^1^Department of Neurosurgery, Brigham and Women’s Hospital, Harvard Medical School, Boston, MA, United States; ^2^Center for Pituitary Tumor Surgery, Department of Neurosurgery, The First Affiliated Hospital, Sun Yat-sen University, Guangzhou, China; ^3^Wuhan School of Clinical Medicine, Southern Medical University, Wuhan, China; ^4^Department of Psychiatry, Brigham and Women’s Hospital, Harvard Medical School, Boston, MA, United States; ^5^Institute for Technology in Psychiatry, McLean Hospital, Harvard Medical School, Belmont, MA, United States; ^6^Department of Radiology, Brigham and Women’s Hospital, Harvard Medical School, Boston, MA, United States

**Keywords:** sex effect, presurgical language mapping, brain tumor, functional MRI (fMRI), functional connectivity, supplementary motor area (SMA), language processing

## Abstract

Differences between males and females in brain development and in the organization and hemispheric lateralization of brain functions have been described, including in language. Sex differences in language organization may have important implications for language mapping performed to assess, and minimize neurosurgical risk to, language function. This study examined the effect of sex on the activation and functional connectivity of the brain, measured with presurgical functional magnetic resonance imaging (fMRI) language mapping in patients with a brain tumor. We carried out a retrospective analysis of data from neurosurgical patients treated at our institution who met the criteria of pathological diagnosis (malignant brain tumor), tumor location (left hemisphere), and fMRI paradigms [sentence completion (SC); antonym generation (AG); and resting-state fMRI (rs-fMRI)]. Forty-seven patients (22 females, mean age = 56.0 years) were included in the study. Across the SC and AG tasks, females relative to males showed greater activation in limited areas, including the left inferior frontal gyrus classically associated with language. In contrast, males relative to females showed greater activation in extended areas beyond the classic language network, including the supplementary motor area (SMA) and precentral gyrus. The rs-fMRI functional connectivity of the left SMA in the females was stronger with inferior temporal pole (TP) areas, and in the males with several midline areas. The findings are overall consistent with theories of greater reliance on specialized language areas in females relative to males, and generalized brain areas in males relative to females, for language function. Importantly, the findings suggest that sex could affect fMRI language mapping. Thus, considering sex as a variable in presurgical language mapping merits further investigation.

## Introduction

Image-guided neurosurgery, including presurgical functional mapping with functional magnetic resonance image (fMRI), is increasingly used by neurosurgeons to perform safer, more precise, and less invasive brain surgery (Golby, 2015). Accurate presurgical planning is critical for maximizing tumor removal and maximally preserving eloquent cortices that support critical brain functions, such as motor control and language (Tharin and Golby, 2007; Silva et al., 2018). Presurgical language mapping can facilitate the neurosurgeon’s decision to limit the extent of tumor resection in order to preserve function; or give the neurosurgeon more confidence to proceed with tumor removal, particularly when the tumor is in close proximity to classical language regions.

For presurgical mapping of language function, fMRI based on blood oxygen level-dependent (BOLD) signal changes during language task performance versus rest has been widely adopted (Bookheimer, 2007). A variety of fMRI paradigms are available for presurgical brain mapping of language function. The American Society of Functional Neuroradiology (ASFNR) recommends for adults a three-task battery consisting of sentence completion (SC) and silent word generation, followed by either rhyming, object naming, or passive story listening (Black et al., 2017). Our recent retrospective study comparing three language tasks used for presurgical planning [SC, antonym generation (AG), and auditory naming] demonstrated in a large cohort of brain lesion patients that SC relative to the other tasks elicited a greater extent of activation within the posterior language areas of the dominant hemisphere, and was therefore considered more effective for determining language laterality in the brain (Unadkat et al., 2019). Despite the progress in evaluating language paradigms for fMRI presurgical mapping in brain tumor patients (Tie et al., 2014, 2015; Black et al., 2017), the influence of demographic factors such as sex has not yet been systematically evaluated.

The study of sex differences in language processing can be dated back to the late 1950s. Early work claimed that females outperform males in terms of general language abilities from childhood and through adulthood (Anastasi, 1958). However, subsequent work suggested that females outperform males specifically in verbal fluency tasks, whereas males outperform females in visuospatial tasks including mental rotation (Herlitz et al., 1999). Shaywitz and colleagues were among the first to employ fMRI to study sex differences in language processing in the brain (Shaywitz et al., 1995). Their findings suggested that females’ language networks are more bilateral, while males’ are more left-lateralized. A number of later fMRI studies supported the idea that the better performance of females relative to males in verbal fluency tasks is related to a greater dependence on declarative memory for the processing of complex language forms in females (Kansaku et al., 2000; Phillips et al., 2001; Becker et al., 2007). In contrast, males relative to females may rely more on procedural memory for the processing of complex linguistic forms (Ullman, 2001; Becker et al., 2007).

Whether there exist sex differences in performance of language paradigms used for presurgical evaluation is unknown. This issue is important because sex differences in presurgical language mapping could affect the accuracy of this procedure, and subsequent language outcomes and interventions. In the context of presurgical language mapping, it is also important to consider whether there are sex differences in neurosurgical patients over and beyond the differences observed in neurologically healthy individuals (Coates, 2015). In brain tumor patients, sex differences in language mapping could reflect influences of sex on language processing *per se* (as seen in the general population), as well as influences of sex on brain tumor location, resilience to the disease, and neuroplasticity, that could affect language organization and function in these patients (Hyer et al., 2018; Ostrom et al., 2018a). Evidence for sex differences in brain tumor patients comes from epidemiological studies showing that the male to female incidence ratio of glioblastoma, the most common malignant brain tumor (MBT), is 1.6:1 in the United States (Ostrom et al., 2018a), and the survival rate is better in females compared to males with glioblastoma (median survival of 22.6 versus 15.9 months) (Ostrom et al., 2018b).

To examine the possibility of sex influences on presurgical language mapping, we retrospectively analyzed language task fMRI activation maps and resting-state (RS) fMRI connectivity maps in 47 neurosurgical patients with MBTs who met the study inclusion criteria. The study was restricted to patients with high-grade brain tumors to reduce confounding effects of functional reorganization (that are more common in patients with low-grade brain tumors and longer survival rates) (Ghinda and Duffau, 2017).

## Materials and Methods

### Participants

We retrospectively analyzed data from all brain tumor patients who underwent both task-based fMRI language mapping and rs-fMRI at Brigham and Women’s Hospital between September 1^st^, 2012 and August 1^st^, 2018. The inclusion criteria were: (1) diagnosis of MBT, defined by a final pathology diagnosis of high-grade brain tumor (WHO III-IV) according to the 2016 World Health Organization Classification of Tumors of the Central Nervous System (Louis et al., 2016); (2) brain tumor located in the left hemisphere; (3) presurgical fMRI assessment with SC and AG tasks, and resting state (RS). In total, 47 patients with MBTs were found eligible for inclusion in the study, 22 females (54.5 ± 10.1 years) and 25 males (57.6 ± 17.3 years). The MBTs included primary and recurrent glioblastoma, anaplastic astrocytoma, primary and recurrent anaplastic oligodendrogliomas, and metastases. All the patients were native English speakers, and none were fluent in a second language. The patients’ demographic information is reported in Table 1, including handedness determined by the Edinburgh Handedness Inventory (EHI) (Oldfield, 1971) and language dominance assessed by clinical fMRI reports (consisting of a qualitative assessment of the activation lateralization during language tasks in the entire brain, performed by a neuroradiologist).

**TABLE 1:** Demographic and clinical characteristics of the patients.

	Sex		*p*-Value
	Female (*n* = 22)	Male (*n* = 25)	
Age (mean ± SD, years)	54.5 ± 10.1	57.6 ± 17.3	0.135^a^
Handedness, number of patients (median EHI value)			0.567^b^
*Left (EHI* < *−25)*	3 (*−*50)	4 (*−*75)	
*Right (EHI* > + *25)*	18 (100)	18 (97.5)	
*Ambidextrous (−25* < *EHI* < + *25)*	0	2 (0)	
Tumor location (number of patients)			0.343^b^
*Frontal*	5	6	
*Temporal*	9	13	
*Parietal*	3	1	
*Insula*	0	2	
*Occipital*	1	0	
*Frontotemporal*	2	0	
*Frontoparietal*	1	2	
*Frontoinsula*	0	1	
*Temporoparietal*	1	0	
Tumor classification			0.559^b^
*GBM (recurrent GBM)*	15 (7)	20 (8)	
*Anaplastic astrocytoma*	2	3	
*AO (recurrent AO)*	3 (2)	1 (0)	
*Metastasis*	2	1	
Tumor volume (median, IQR, cm^3^)	23.1 (8.5–34.2)	19.2 (9.9–48.8)	0.874^a^
Language dominance			0.611^b^
*Left*	17	22	
*Right*	2	1	
*Bilateral*	3	2	
Seizures, number of patients			0.679^b^
*No*	17	18	
*Unknown*	5	7	
AED, number of patients			0.291^b^
*Yes*	10	17	
*No*	8	5	
*Unknown*	4	3	

*^*a*^Mann–Whitney *U*-test, ^*b*^Chi-Squared Test of sex differences. AED, anti-epileptic drug; AO, anaplastic oligodendrogliomas; EHI, Edinburgh Handedness Inventory; GBM, glioblastoma; IQR, interquartile range; SD, standard deviation. Language dominance was assessed by the clinical fMRI report (consisting of a qualitative assessment of the activation lateralization during language tasks in the entire brain, performed by a neuroradiologist). Seizures were tallied in the period of 24 h preceding the scan and the period of the scan.*

All the procedures of this study were in accordance with the Declaration of Helsinki and approved by the Partners Institutional Review Board. The study protocol was fully explained to the patients prior to the acquisition of fMRI, and then patients provided written informed consent for research use of their imaging and clinical data.

### Image Acquisition

Prior to MRI scanning, the patients underwent standard MR screening and were instructed on how to perform the language tasks. Patients did not receive fMRI (and were excluded from this study) if they had any contraindications to MR scanning or failed to understand the task instructions.

Structural and functional MRI was performed on a 3.0 Tesla Siemens scanner (Siemens Trio, Verio, Skyra, and Prisma Systems, Munich, Germany; see details of scanners in Supplementary Material S1) with a 20-channel head coil. Participants were placed in a supine position with their head fixed by positioning cushions to minimize head motion artifacts. BOLD fMRI was acquired using single-shot T2^∗^-weighted gradient echo planar imaging (EPI) with the following parameters: repetition time (TR) = 2000 ms, echo time (TE) = 30 ms, flip angle = 85°, Matrix = 64 × 64, field of view (FOV) = 220 mm × 220 mm, voxel size = 3.44 × 3.44 × (4.0 or 5.0) mm^3^, 24 or 32 axial slices, ascending interleaved sequence.

A high resolution T2 weighted image was also acquired for the clinical fMRI report. Structural MRI was performed for surgical planning as clinically indicated, including a high resolution T1 weighted anatomical image with contrast (gadolinium) administration (axial 1 mm slices). The structural images were used for spatial co-registration and normalization of the fMRI.

### Stimulus Paradigm

The fMRI language tasks consisted of SC and AG. The SC task alternates between a high-level control condition in which the patient is shown non-pronounceable letter strings, and an experimental condition in which coherent sentences missing a single word are shown and the patient is instructed to complete the sentence silently. Six cycles of the control followed by the SC conditions are presented for a total duration of 4 min. In the AG task, the patient is presented with a single word, and is instructed to silently think of an antonym to that word. This condition alternates with a control condition in which a crosshair is shown. Six cycles of the AG and control conditions are presented for a total duration of 5 min. The stimuli were presented using a visual and audio stimulation system (Nordic Neurolab, Bergen, Norway). During the rs-fMRI scan, participants were instructed to keep still with their eyes closed for a duration of 4 (6 patients), 5 (23 patients), or 7 min (18 patients).

### Brain Tumor Mapping

Brain tumor mapping was performed to defend against the possibility of a confounding effect of sex on tumor location. In each patient, we defined a conservative brain tumor mask that included a prior resection cavity and surrounding edema, as determined by both high density and proximity to the tumor boundary in the T1-enhancement MRI. Brain tumor masks were semi-automatically drawn on the T1-enhancement MRI by a post-doctoral research fellow and reviewed by a clinical fellow using the Brainlab Surgery Platforms and Software^1^ and 3D Slicer^2^. Both investigators were blinded to the patients’ clinical information. The brain tumor volume was calculated in each patient based on the brain tumor mask. The transformation matrix created in the normalization process (see below fMRI data preprocessing) was applied to all brain tumor images for registration to the standard MNI-152 template (1 mm isotropic voxels) using FLIRT (FMRIB’s Linear Image Registration Tool^3^).

### fMRI Preprocessing

fMRI analysis was performed using FSL (FMRIB Software Library Version 6.00^4^) (Jenkinson et al., 2012). The first five dummy scan volumes were discarded to allow for stabilization of the BOLD signal. fMRI pre-processing included (1) head motion correction using Motion Correction FLIRT (MCFLIRT) (Jenkinson et al., 2012); (2) non-brain tissue removal of functional images using the default Brain Extraction Tool (BET) (Smith, 2002); (3) indirect normalization; (4) non-linear spatial smoothing using a Gaussian kernel with a full width at half maximum (FWHM) value of 6 mm; (5) high-pass filtering to remove linear drifts and low frequency noise. For rs-fMRI, additional preprocessing steps were performed, including denoising using CompCor strategy implemented in CONN Toolbox^5^ (Behzadi et al., 2007), and bandpass filtering (0.008–0.09 Hz).

Normalization of brain images into a standard space is necessary for comparison across individuals but challenging in the presence of extensive brain pathology related to a tumor. For example, it may be difficult to determine the boundary between gray matter and the surrounding cerebral spinal fluid filled spaces in highly progressive MBT. We employed an optimized brain extraction algorithm for brains with pathological structures which has shown better performance on brain tissue extraction than other available tools (Lutkenhoff et al., 2014). To facilitate group level analyses, the fMRI images were indirectly normalized to the Montreal Neurological Institute (MNI) space. A binary lesion mask was first created and used to weight the linear registration of the functional images to the T1 images, and subsequently to the standard MNI-152 template using cost function masking (Brett et al., 2001; Andersen et al., 2010) implemented with FMRIB’s Linear Image Registration Tool (FLIRT v6.0) (Jenkinson et al., 2002, 2012). These steps are available in the FMRI Expert Analysis Tool (FEAT v6.0) module in the FSL package. An example of the optimized normalization procedure in one patient with a large brain tumor is shown in Supplementary Material S2.

### Task-Based fMRI Analysis

The first-level fMRI analysis was carried out using the general linear model (GLM) module of FSL. The resulting z-maps were non-parametrically thresholded using a cluster threshold of *Z* > 3.1 (*p* < 0.05, corrected for multiple comparisons). Motion signals were discarded from fMRI by regressing out a confound matrix consisting of the 6 motion parameters.

For the second-level analysis (fixed-effects), a two-way ANCOVA (sex × language task) was performed using GLM and covariates of age, tumor volume, tumor location, handedness, and scanner type. The tumor location was coded according to the lobe (frontal, temporal, insula, parietal, occipital, frontotemporal, frontoparietal, frontoinsula, and temporoparietal). The continuous (age and tumor volume) and categorical (tumor location, handedness scores, and scanner type) covariates were mean-centered across all participants by subtracting the overall mean value from each covariate. The resulting z-maps were non-parametrically thresholded using a cluster threshold of *Z* > 3.1 (*p* < 0.05, corrected for multiple comparisons).

Language lateralization was assessed for each task and for the combination of tasks using a laterality index (LI) calculated based on the amplitude of activation in the left and right hemispheres in anatomically-defined language regions-of-interest (ROIs) (Rolls et al., 2015). The anterior language ROI included the pars opercularis, pars triangularis, and pars orbitalis of the inferior frontal gyrus (IFG). The posterior language ROI included the posterior aspect of the superior and middle temporal gyri (STG/MTG), angular gyrus (AG), and supramarginal gyrus (SMG) [Talairach y coordinates −28 to −59 (Ochiai et al., 2004; Liebenthal et al., 2014)]. All ROIs were then inverted into patient-individual space using FSL command-line utilities. The LI calculation was adjusted to correct for the possibility that language ROIs were affected by the lesion. In each patient, the areas of overlap between the lesion and the language ROIs were masked and non-lesioned language ROIs were created. The lesion-free anterior and posterior language ROIs, and their combination (anterior + posterior), in each hemisphere, were used to compute the LI using a Lesion-Adjusted Formula (Dietz et al., 2016), as follows:

$$\text{LI}=\frac{\frac{\#\,L\,e\,f\,t\,A\,c\,t\,i\,v\,e\,V\,o\,x\,e\,l\,s}{\mathrm{Left}\,\mathrm{Nonlesioned}\,\mathrm{Voxels}}-\frac{\#\,R\,i\,g\,h\,t\,A\,c\,t\,i\,v\,e\,V\,o\,x\,e\,l\,s}{\mathrm{Right}\,\mathrm{Nonlesioned}\,\mathrm{Voxels}}}{\frac{\#\,L\,e\,f\,t\,A\,c\,t\,i\,v\,e\,V\,o\,x\,e\,l\,s}{\mathrm{Left}\,\mathrm{Nonlesioned}\,\mathrm{Voxels}}+\frac{\#\,R\,i\,g\,h\,t\,A\,c\,t\,i\,v\,e\,V\,o\,x\,e\,l\,s}{\mathrm{Right}\,\mathrm{Nonlesioned}\,\mathrm{Voxels}}},$$

where active voxels were tallied only within the non-lesioned portion of each ROI. Language lateralization was defined as left (LI ≥ 0.2), right (LI ≤−0.2), or bilateral (−0.2 < LI < 0.2) (Binder et al., 1996; Unadkat et al., 2019).

### Resting-State Functional Connectivity (RSFC) Analysis

The seed-to-voxel RSFC analysis across the whole brain was performed using the CONN Toolbox v.18.a http://www.nitrc.org/projects/connand SPM12 (Welcome Trust Centre for Neuroimaging, UCL, United Kingdom) running in MATLAB R2018a (MathWorks, Inc., Natick, MA, United States).

The supplementary motor area (SMA) was selected as the seed for functional connectivity analysis because this area showed a main effect of sex in the ANCOVA and none of the participants had a tumor infiltrating this area. The effect of sex on SMA functional connectivity was corrected for multiple comparisons using the seed-level false discovery rate (FDR) approach with a significance threshold of *p* < 0.05 (two-sided). The same covariates were used in the RSFC analysis as in the task-based fMRI analysis.

### Statistical Analysis

The statistical analyses of clinical characteristics were performed in an open-source statistical software package, JASP (JASP Version 0.92.0, University of Amsterdam, Netherlands^6^) (Marsman and Wagenmakers, 2017). Group differences in continuous clinical variables were determined using the non-parametric Mann–Whitney *U*-test or the two-sample Student’s *t*-test, depending on the distributions of the variables. Group differences in categorical variables were determined using the Pearson χ^2^ test. The statistical significance threshold was set at *p* < 0.05 (two-tailed) for all the analyses.

## Results

### Clinical Characteristics

The patients’ demographic and clinical characteristics are reported in Table 1. The number of patients with left, right, and ambidextrous handedness, as determined by EHI, was 7, 36, and 2, respectively (two patients’ EHI information was missing). The number of patients with left, right, and bilateral hemispheric dominance for language, as assessed by clinical fMRI, was respectively, 39 (33 right-handed, 4 left-handed, and 1 ambidextrous), 3 (1 right-handed, 2 left-handed), and 5 (2 right-handed, 1 left-handed, and 1 ambidextrous). There were no significant differences between the female and male patients in the demographic and clinical characteristics, including age (*p* = 0.135), handedness (*p* = 0.567), tumor location (*p* = 0.343), pathological classification (*p* = 0.559), tumor volume (*p* = 0.874), and language dominance based on the fMRI clinical report (*p* = 0.611).

**FIGURE 1:**
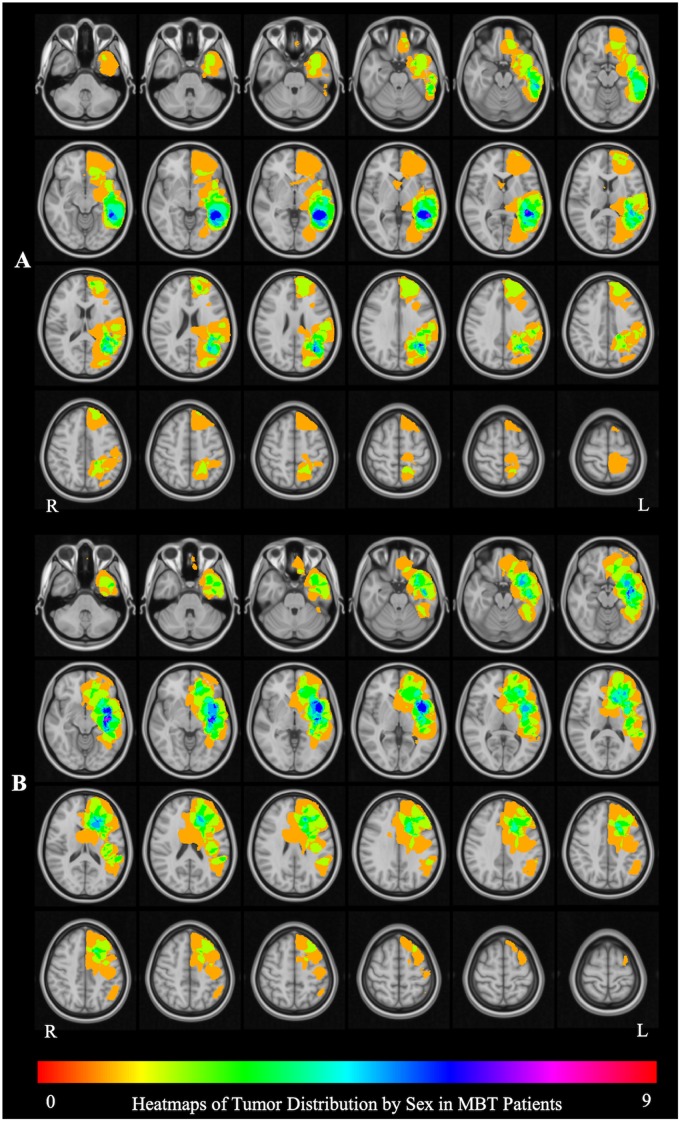
Brain tumor location by sex. **(A)** The aggregate map of brain tumor location in male patients. The peak tumor locations are in in the medial aspect of the left middle temporal [9/25 patients; peak coordinate (x, y, z): –34, –15, –10] and inferior frontal (6/25 patients; peak coordinate: –28, 25, 0) cortex. **(B)** The aggregate map of tumor location in female patients. The peak tumor location is in the lateral aspect of the left posterior middle temporal gyrus (MTG) (7/22 patients; peak coordinate: –52, –42, 1). The heat maps represent the number of patients with a tumor infiltrating the brain at each location.

The aggregate map of brain tumor location in male patients (Figure 1A) shows that the peak tumor locations in males were in the medial aspect of the left middle temporal [9/25 patients; peak coordinate (x, y, z): −34, −15, −10] and inferior frontal (6/25 patients; peak coordinate: −28, 25, 0) cortex, whereas the peak tumor location in females was in the lateral aspect of the left posterior middle temporal gyrus (MTG) (7/22 patients; peak coordinate: −52, −42, 1) (Figure 1B). The tumor location was therefore included as a covariate in all the fMRI analyses, and the interpretation of the fMRI results was constrained in the brain areas differentially affected by tumor in the male and female patients.

### Whole Brain Activation

**FIGURE 2:**
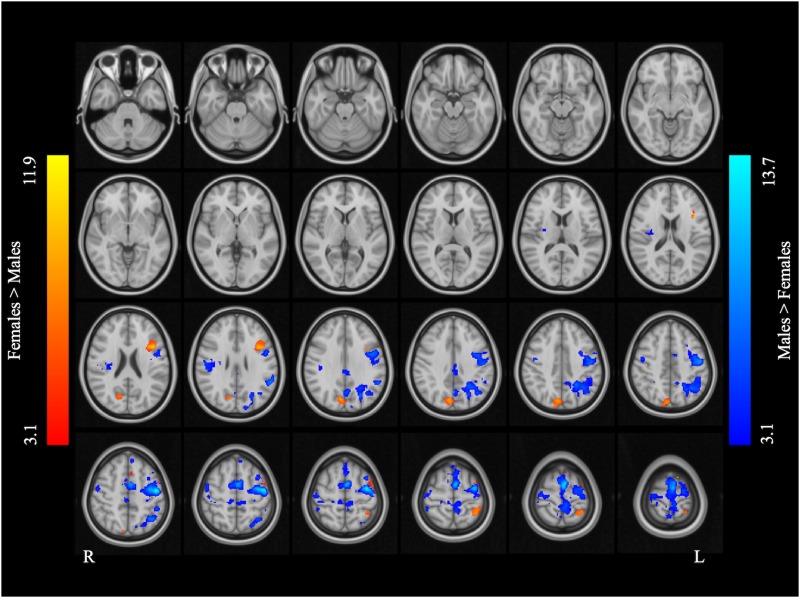
Main effect of sex across the two language tasks. The areas rendered by the red-yellow color scale indicate greater activation in females compared to males, whereas the areas rendered by the blue-light blue color scale indicate greater activation in males relative to females (Z-score > 3.1, cluster size > 200 voxels, corrected *p* < 0.05).

Figure 2 and Table 2 report the main effect of sex across language tasks. Compared with the females, the males showed greater activation in the left SMA, bilateral precentral gyrus, left inferior parietal lobule (IPL), and right Rolandic operculum. Compared with the males, the females showed greater activation in small volumes of the right cuneus, left inferior opercular and triangular parts of the IFG, and left superior parietal lobule (SPL).

**TABLE 2:** Main effect of sex across the two language tasks.

Cluster size	Brain regions	L/R	BA	Peak MNI coordinates			*Z*-values
				x	y	z	
**Female > Male**							
403	Cuneus/precuneus	R	7	8	−78	38	7.62
286	Pars triangularis/opercularis	L	44	−42	14	22	9.26
230	Superior parietal lobule	L	7	−36	−48	62	6.49
**Male > Female**							
7542	SMA	L	6	−2	−2	66	12.17
	Precentral gyrus	L	6	−46	−10	50	13.66
	Post-central gyrus	L	7	−6	−46	66	6.42
	Precentral gyrus	R	6	46	−6	42	6.28
	Superior frontal gyrus	L	8	−6	42	48	5.96
2303	Inferior parietal lobule	L	39/7	−42	−58	48	8.41
458	Rolandic operculum	R	40	54	−24	24	6.75
286	Posterior cingulate gyrus	R	23	0	−30	34	6.75

*Z-score > 3.1, cluster size > 200 voxels, corrected *p* < 0.05.BA, Brodmann area; MNI, Montreal Neurological Institute; SMA, supplemental motor area; L, left; R, right.*

**FIGURE 3:**
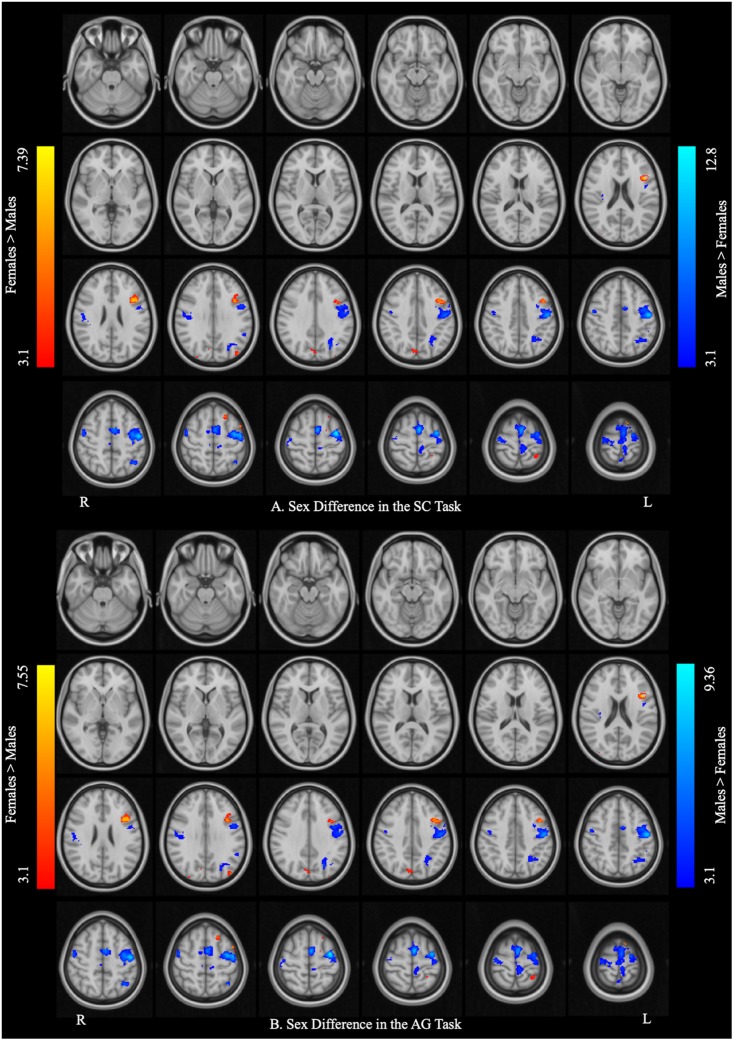
Sex differences in each language task. **(A)** Sex difference in the sentence completion (SC) task. **(B)** Sex difference in the antonym generation (AG) task. The areas rendered by the red–yellow color scale indicate greater activation in females relative to males, whereas the areas rendered by the blue-light blue color scale indicate greater activation in males relative to females (Z-score > 3.1, cluster size > 150 voxels, corrected *p* < 0.05).

Figure 3 and Table 3 report the effect of sex in each language task. In the SC task, the males showed greater activation than the females in the bilateral precentral gyrus, left SMA, and left IPL, and the females showed greater activation than the males in the right precuneus, left pars opercularis, and left SPL. In the AG task, the males showed greater activation in the left precentral gyrus and SMA, left precuneus, left IPL, and right Rolandic operculum, while the females showed greater activation in the right precuneus, left SPL, and left pars triangularis. The main effect of language task across sex and the interaction between task and sex are reported in Supplementary Materials S3, S4, respectively.

**TABLE 3:** Sex differences in each language task (*post hoc analysis).*

Task	Cluster size	Brain regions	L/R	BA	Peak MNI coordinates			*Z*-values
					x	y	z	
**SC task**	**Female > Male**							
	691	Precuneus	R	7	8	−76	38	4.03
	450	Pars opercularis	L	44	−40	14	18	7.39
	364	Superior parietal lobule	L	40	−30	−48	68	3.88
	**Male > Female**							
	5069	Precentral gyrus	L	6	−36	−6	60	12.82
		SMA	L	6	−2	1	65	10.58
		Precentral gyrus	R	6	36	−18	68	5.47
	1221	Inferior parietal lobule	L	7	−38	−58	50	6.06
	413	Rolandic operculum	R	40	54	−14	24	4.90
**AG task**	**Female > Male**							
	1202	Precuneus	R	7	6	−78	42	7.55
	391	Superior parietal lobule	L	40	−40	−42	62	6.64
	426	Pars triangularis	L	45	−42	14	22	6.47
	**Male > Female**							
	5068	Precentral gyrus	L	4	−44	−10	54	9.36
		SMA	L	6	−4	−4	70	8.53
	1439	Precuneus	L	31	0	−64	32	7.43
		Angular gyrus/IPL	L	39/40	−44	−58	46	7.39
	168	Rolandic operculum	R	40	54	−24	24	5.02

*Z-score > 3.1, cluster size > 150 voxels, corrected *p* < 0.05.AG, antonym generation; BA, Brodmann area; MNI, Montreal Neurological Institute; SC, sentence completion; SMA, supplemental motor area; L, left; R, right.*

### Language Lateralization

Table 4 reports the lesion-adjusted LI for males and females in the language ROIs for each task and the combination of tasks. Only the posterior language ROI in the SC task showed sex differences in LI (*p* = 0.015). This ROI was significantly more left lateralized in the males.

**TABLE 4:** Lateralization index results within the language ROI in the dominant hemisphere.

Task		Laterality			*p*-Value*
		Left	Bilateral	Right	
**Anterior ROI**					
SC	Female	15	6	1	0.318
	Male	19	3	3	
AG	Female	14	7	1	0.552
	Male	12	11	2	
SC + AG	Female	15	5	2	0.988
	Male	17	6	2	
**Posterior ROI**					
SC	Female	11	6	5	0.015
	Male	22	1	2	
AG	Female	11	8	3	0.452
	Male	17	6	2	
SC + AG	Female	12	6	4	0.412
	Male	18	5	2	
**Global**					
SC	Female	15	4	3	0.431
	Male	21	2	2	
AG	Female	11	9	2	0.645
	Male	15	7	3	
SC + AG	Female	12	8	2	0.298
	Male	19	5	1	

*Non-parametric Mann–Whitney *U*-test. AG, antonym generation; SC, sentence completion; ROI, region-of-interest.

### SMA-Based Functional Connectivity

**FIGURE 4:**
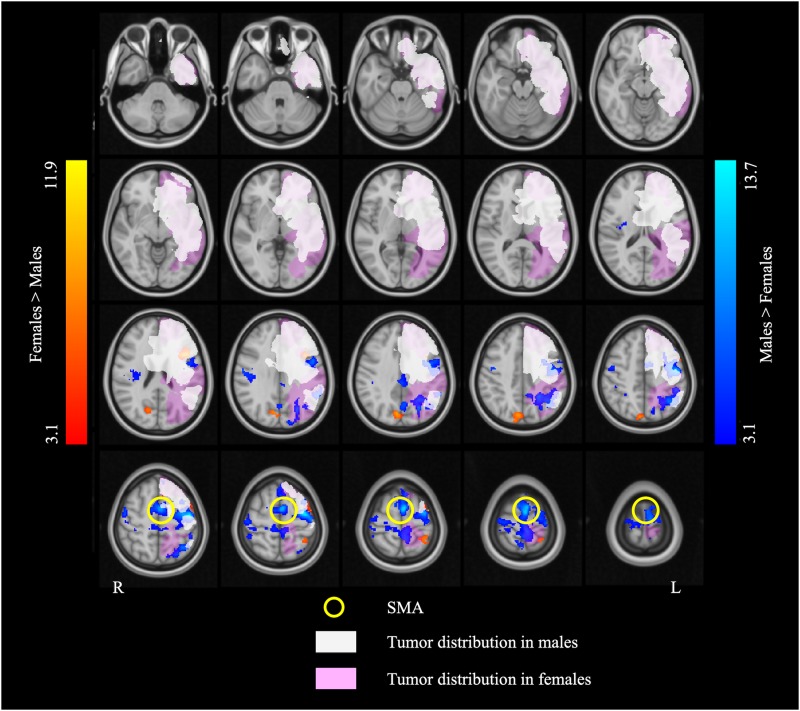
The overlap map of fMRI activation and brain tumor distribution. The white and pink overlays represent the areas affected by brain tumor in the males and the females, respectively. The blue and red overlays represent the fMRI activation in the two language tasks in the males and the females, respectively. Note that the supplementary motor area (SMA) (indicated by the yellow circles) is completely outside of the areas afflicted by tumor in both males and females.

**FIGURE 5:**
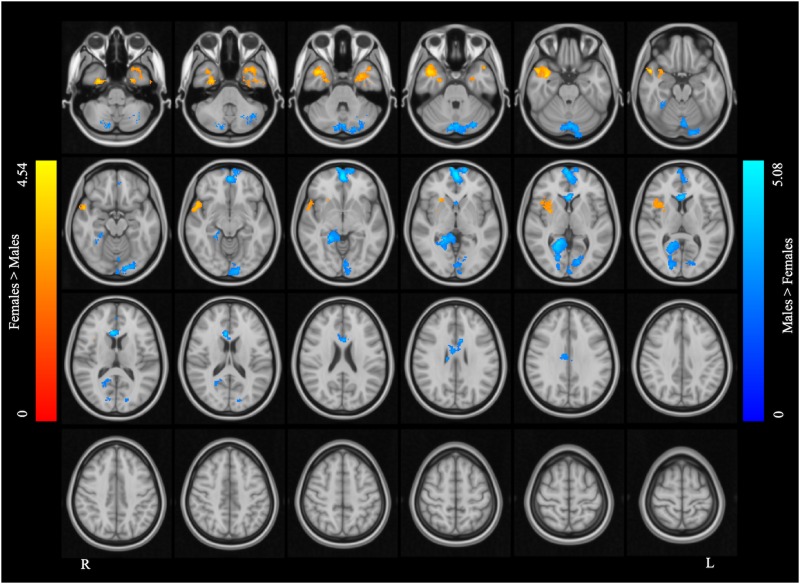
Supplementary motor area resting state functional connectivity maps. The blue color rendered areas show increased FC with the SMA in males, and the red color rendered areas show increased FC with the SMA in females (cluster size > 200 voxels, corrected *p* < 0.05).

The left SMA was selected as the seed for functional connectivity analysis because this area showed sex differences in the performance of language task-based fMRI, and it was well outside the areas afflicted by tumor in all participants (Figure 4). The results of the functional connectivity analysis are reported in Figure 5 and Table 5. The males compared to the females showed increased functional connectivity between the left SMA and the left cerebellum, left and right ventromedial prefrontal cortex (vmPFC), right lingual gyrus, and anterior cingulate cortex (ACC) (Figure 5, cold color rendered areas). Compared to the males, the females showed increased functional connectivity between the left SMA and the bilateral inferior TP, and right putamen (Figure 5, warm color rendered areas).

**TABLE 5:** Results of seed-based correlation analysis using SMA as seed region.

Cluster size	Brain regions	L/R	BA	Peak MNI coordinates			Effect size	*t*-Values

				x	y	z		
**Female > Male**								
1130	Inferior temporal pole	R	20	28	−8	−44	0.24	5.47
595	Inferior temporal pole	L	20	−24	−6	−42	0.21	4.30
287	Putamen	R	/	30	2	2	0.23	3.78
**Male > Female**								
1995	Cerebellum	L	/	10	−82	−30	0.23	4.73
1027	Ventromedial prefrontal cortex	L	10	0	58	−4	0.24	5.31
	Ventromedial prefrontal cortex	R	10	2	64	−2	0.24	5.31
1235	Lingual gyrus	R	30	16	−48	4	0.25	4.43
609	ACC	R	24	6	24	12	0.24	5.38

*Cluster size > 200 voxels, corrected *p* < 0.05. ACC, anterior cingulate cortex; BA, Brodmann area; MNI, Montreal Neurological Institute; L, left; R, right.*

## Discussion

In this retrospective study, the activation patterns induced by SC and AG tasks used for presurgical language mapping were compared between male and female patients with MBT. The male (*n* = 0.25) and female (*n* = 22) patients were matched on all the demographic and clinical variables. Although brain tumor location overlapped considerably between the male and female patients, it involved the medial aspect of the left middle temporal and inferior frontal cortex in more male patients, and the lateral aspect of the left posterior MTG in more female patients. Tumor location was therefore included as a covariate in all the fMRI analyses, and the interpretation of fMRI results was constrained in the brain areas differentially affected by tumor in the male and female patients. The findings revealed a main effect of sex across language tasks, including in areas spared by lesion in all patients. The males relative to the females showed stronger activation in relatively extensive brain areas, including areas associated with generalized cognitive (i.e., not language-specific) processing (left SMA and IPL, bilateral precentral gyrus). The females relative to the males showed stronger activation in limited brain areas, including areas classically associated with language processing (left IFG). Sex differences were also observed in the RSFC patterns of the left SMA: in males relative to females the left SMA connectivity tended to be stronger with midline areas (ventromedial prefrontal cortices, lingual gyrus, anterior cingulate) and the cerebellum, and in females relative to males it tended to be stronger with lateral temporal areas (bilateral inferior temporal poles).

### Brain Areas Showing Males > Females Activations

Compared to the females, the males showed greater activation in extensive brain areas including the left SMA and IPL, bilateral precentral gyrus, and right Rolandic operculum, across language tasks. The SMA is implicated mainly in motor control (Goldberg, 1985; Tanji, 1994). However, lesion and stimulation studies suggest that the SMA also plays a pivotal role in higher-order cognitive control, including control of speech perception and speech initiation (Krainik et al., 2003; Tourville and Guenther, 2011; Hertrich et al., 2016). Neurosurgical resection involving the SMA may lead to the “SMA syndrome” characterized by contralateral akinesia and mutism (Laplane et al., 1977; Bannur and Rajshekhar, 2000). The left SMA was shown to be recruited during sentence-level (Zacà et al., 2012, 2013; Black et al., 2017) and word-level (Wise et al., 1991; Warburton et al., 1996; Black et al., 2017) language processing. Interestingly, the SMA was found to more frequently be affected by low-grade brain tumors than MBTs (Duffau and Capelle, 2004), consistent with our finding of no tumor infiltration in the SMA in any of the patients. Because the SMA was spared by lesion in the present patient sample, the finding of greater left SMA activation in males relative to females may specifically be attributed to greater engagement of this area in language processing in the males.

Areas in the bilateral precentral gyrus have also been shown to support motor aspects of language function (Price, 2000, 2010). Damage to the left precentral gyrus is associated with speech production deficits (Mori et al., 1989; Itabashi et al., 2016). A meta-analysis of studies examining brain volume and density has previously reported a significantly larger gray matter volume in the left precentral gyrus in males relative to females (Ruigrok et al., 2014). In the present study, the effect of sex on activation in the left precentral gyrus was extensive, also covering part of the right precentral gyrus, and could not be explained strictly by a sex difference in tumor location (because the incidence of tumor in the left precentral gyrus was higher in the males).

The left IPL (Brodmann area 40) is part of a dorsal auditory-motor stream implicated in speech perception and phonological processing (Liebenthal et al., 2013; Hickok and Poeppel, 2015; Liebenthal and Möttönen, 2018). However, in this area, the finding of greater activation in the males may be confounded by the greater occurrence of lesions in the females.

Taken together, the findings of stronger activation of the left SMA and bilateral precentral gyrus are consistent with the possibility of greater engagement of a network of areas associated with motor and speech control during language processing in males relative to females.

### Brain Areas Showing Females > Males Activations

Compared to the males, the females showed greater activation in the left IFG, left SPL, and right cuneus/precuneus in both tasks. The left pars opercularis in the IFG is thought to be an important node of the articulatory network involved in speech production and phonological processing, as well as syntactic processing (Geschwind, 1970; Ojemann, 1979; Poeppel, 1996; Binder et al., 2009). Structural brain imaging studies in healthy adults have revealed larger gray matter volumes in the left IFG (opercularis and triangularis) in females compared to males (Im et al., 2006; Ruigrok et al., 2014; Kurth et al., 2017). Functional brain imaging studies in healthy adults have also indicated that females show greater activation in the IFG than males during emotional speech perception (Schirmer et al., 2004) and mental rotation (Hugdahl et al., 2006) tasks. Our findings are consistent with these previous reports suggesting sex differences in the function of the left IFG. However, the present findings are limited by the fact that the male patients had a higher occurrence of tumors in medial aspect of the left IFG, which could have contributed to the finding of greater left IFG activation in the females.

The region of the cuneus and precuneus has been associated with high-level cognitive functions including episodic memory retrieval (Lundstrom et al., 2003; Sadigh-Eteghad et al., 2014), visuospatial imagery (Cavanna and Trimble, 2006), self-reflection, and consciousness (Vogt and Laureys, 2005; Cavanna and Trimble, 2006). Studies in patients with a disorder affecting language, such as primary progressive aphasias (Rohrer et al., 2010) and schizophrenia (Mashal et al., 2014), support the notion that this area may be involved in phonological processing. The common finding of increased activation of the precuneus and posterior cingulate in tasks emphasizing semantic processing has been tied to a role for this general area (similar to that of the parahippocampal gyrus) as an interface between the semantic memory and episodic retrieval systems by virtue of strong connectivity with the hippocampus (Binder et al., 2009). In the present study, the greater activation of the precuneus in the females relative to the males could reflect a greater reliance on episodic memory for semantic processing in the females.

### Increased SMA RSFC in Males Versus Females

We selected the left SMA as the seed for functional connectivity analysis because this area showed sex differences, and it was well outside the area afflicted by tumor in all participants (Figure 4). While the SMA is classically associated with motor function, as evident from the fact that brain surgery in this region can lead to the SMA syndrome consisting of contralateral transient akinesia, mutism, and post-operative motor and speech production deficits (Laplane et al., 1977; Bannur and Rajshekhar, 2000; Oda et al., 2018), there is also evidence to suggest that this area supports certain receptive aspects of language, such as inner speech during language encoding (Hertrich et al., 2016), lexical disambiguation (Chee et al., 1999), syntax and prosody integration (Kotz and Schwartze, 2010), and context-tracking (Bradley et al., 2013). The purpose of the RSFC analysis was to examine the possibility of sex-specific functional connectivity of the left SMA with distributed brain areas supporting language.

In the males relative to the females, increased RSFC was seen between the left SMA and the bilateral vmPFC, right ACC and lingual gyrus, and left cerebellum. The role of the vmPFC is not well-understood: this area has been suggested to participate in high-order mental tasks, such as decision making and assessment of one’s own mental and emotional state (Ramnani and Owen, 2004; Gilbert et al., 2006; Koechlin and Hyafil, 2007; Koechlin, 2011). The ACC, directly structurally connected to the SMA via several short U-fibers (Vergani et al., 2014), is also thought to be involved in high-order functions such as conflict monitoring (Botvinick et al., 2004), cognitive control (Bush et al., 2000), reward-based decision making (Apps et al., 2016; Rigney et al., 2018), and serving as an interface between emotion and cognition (Allman et al., 2001).

The lingual gyrus, in contrast, is a part of the visual cortex involved in the processing of visual features including letters and visual words (Machielsen et al., 2000). Structurally, males have been shown to have larger GMV in the lingual gyrus (Lotze et al., 2019), and this may contribute to the sex difference in the SMA-lingual RSFC pattern observed here. The SMA is also not directly structurally connected with the lingual gyrus, such that the functional connectivity between the two areas may reflect the integration of distinct brain networks (Sporns, 2013).

Overall, the findings are consistent with the possibility that in males relative to females, the functional connectivity of the left SMA was stronger with areas implicated in non-linguistic cognitive processing and control. This domain-non-specific perceptual and cognitive functional network may contribute more significantly to ongoing mental processing in males compared to females.

### Increased SMA RSFC in Females Versus Males

Compared to the males, the females showed increased RSFC between the left SMA and bilateral inferior TP and right putamen.

The TP has been implicated in language processing, based on findings in patients with semantic dementia showing activation in this area during semantic tasks such as object naming and word recognition (Tsapkini et al., 2011; Bonner and Price, 2013; Pascual et al., 2015; Collins et al., 2017). Studies of patients with TP atrophy suggest that patients suffering from semantic variant primary progressive aphasia tend to have the maximal atrophy in the left TP (Collins et al., 2017), whereas patients with right TP atrophy often suffer from social-emotional deficits along with prosopagnosia (Chan et al., 2009; Irish et al., 2013). The putamen is structurally connected with the TP (Middleton and Strick, 1996; Fan et al., 2014) and has also been associated with language processing (Vigneau et al., 2011; Viñas-Guasch and Wu, 2017).

Taken together, the findings are consistent with the possibility that in females relative to males, the functional connectivity of the left SMA was stronger with areas implicated in semantic processing, including areas specialized in the processing of emotional and social concepts. This language and emotion processing functional network may contribute more significantly to ongoing mental processing in females compared to males.

### Language Lateralization

Prior work suggests that activation in the posterior temporal cortex is more left lateralized in males relative to females in a narrative listening block-design task (Kansaku et al., 2000). Consistent with this work, we also found greater left lateralization in the posterior language area (specifically pMTG) in males. The present finding was limited to the SC task, in line with the idea that sex differences in language lateralization may be language paradigm-dependent (Kitazawa and Kansaku, 2005).

### Limitations

The study had several limitations. First, the lesion location differed between males and females in our sample: more females had a lesion involving the lateral aspect of the posterior middle temporal cortex whereas more males had a lesion involving the medial aspect of the middle temporal and inferior frontal cortex. To counter the possibility that tumor effects may have induced a bias in the comparison across sex in our sample, the ANCOVA conducted to assess the effect of sex included tumor location and tumor volume as covariates of no interest. These covariates were found to not have a significant effect (*p* = 0.343 for tumor location and *p* = 0.874 for tumor volume). Nevertheless, the findings of greater activation of the left IFG in the females compared to the males, even though consistent with other literature in healthy subjects, could in the present study be due to the greater occurrence of lesions in this area in the males compared to the females. The greater activation of the left IPL in the males compared the females could be explained by the greater occurrence of tumors in this area in the females compared to the males. Future prospective studies with larger sample sizes should be conducted to overcome the limitations of studying patients with MBTs in variable locations. Furthermore, comparison of sex effects between patients with MBT and neurologically healthy controls may inform the issue of pre-existent versus tumor-related sex differences.

Second, patients were included in this study on the basis of having a MBT in the left hemisphere, and regardless of handedness, because at our institution presurgical language fMRI is ordered for all patients with a left hemisphere MBT. The reasoning is that left hemisphere tumors are likely to be near or within language regions even in individuals with atypical language dominance. In the current sample of 47 patients, three patients (two females) were considered to have right-hemisphere language dominance and five patients (three females) were considered to have bilateral language dominance. To counter the possibility that differences in hemispheric language dominance may have induced a bias in the comparison across sex in our sample, we performed a chi-squared test for the relationship between language dominance and sex. There were no statistically significant differences in the number of patients with atypical language dominance across sex (*p* = 0.611). Future studies with a larger number of patients with atypical handedness could investigate the effect of this factor on the relationship between sex and language function.

Third, formal neuropsychological assessments of speech and language were not performed on the patients. Based on the clinical notes provided by the neurosurgeons, we classified the patients into four language impairment types: (1) normal language function (18 patients, 8 females); (2) receptive aphasia (4 patients, none female); (3) expressive aphasia (21 patients, 12 females); and (4) mixed aphasia (4 patients, 2 females). We performed a chi-squared test for the relationship between language impairment and sex. There were no statistically significant differences in the number of patients with different language impairment type across sex (*p* = 0.214). Therefore, language impairment type determined based on the clinical notes was not included as a variable in the present analyses. However, we anticipate that the degree of impairment in specific aspects of speech and language function could importantly interact with the effect of sex on presurgical language mapping. Thus, future studies investigating the influence of sex on language would benefit from formal assessment of language functions.

Finally, 27 of the patients participating in this study were taking anti-epileptic drugs (AEDs) such as topiramate, carbamazepine, or lamotrigine at the time of scanning. None of the patients had a seizure within 24 h of scanning (Table 1). Taking AEDs and having a seizure may affect language function and language mapping (Aghakhani et al., 2004; Szaflarski and Allendorfer, 2012; Xiao et al., 2018). However, AED use and seizures numbers did not differ significantly between the male and female patients in this study (Chi-Squared Test, *p* > 0.05). Therefore, the risk of AED or seizure effects confounding the effects of sex observed here was considered to be relatively small.

## Conclusion

This study aimed to examine the effect of sex on the pattern of activation and functional connectivity of the brain measured with fMRI presurgical language mapping in patients with a MBT. Across SC and AG tasks, females relative to males showed greater activation in limited areas, including the left IFG classically associated with language processing. In contrast, males relative to females showed greater activation in extended areas beyond the classic language network, including in the SMA and precentral gyrus. The rs-fMRI functional connectivity of the left SMA in the females was stronger with TP areas implicated in semantic and emotion processing, and in the males with several midline areas implicated primarily in cognitive control. These findings are overall supportive of theories of greater reliance on specialized language areas in females relative to males, and generalized brain areas in males relative to females, for language function. Importantly, the findings suggest that sex could affect fMRI language mapping. Thus, considering sex as a variable in presurgical language mapping merits further investigation.

## Data Availability Statement

All datasets generated for this study are included in the article/Supplementary Material.

## Ethics Statement

The studies involving human participants were reviewed and approved by Partners Institutional Review Board. The patients/participants provided their written informed consent to participate in this study.

## Author Contributions

YT, EL, and AG were responsible for the study design, interpreting the data, and critically revising the manuscript. SY drafted the manuscript and performed the data analysis. PJ did the brain tumor segmentation for all patients and AB reviewed all the lesion masks from brain tumor segmentation (both PJ and AB were blind to the clinical data). LR performed MRI scanning and organized the fMRI dataset. AB, MV, and LR assisted in the preparation of the manuscript for publication.

## Conflict of Interest

The authors declare that the research was conducted in the absence of any commercial or financial relationships that could be construed as a potential conflict of interest. The reviewer JS declared a past co-authorship with one of the authors AG to the handling Editor.

## Footnotes

1 https://www.brainlab.com

2 https://www.slicer.org/

3 https://fsl.fmrib.ox.ac.uk/fsl/fslwiki/FLIRT

4 https://fsl.fmrib.ox.ac.uk/fsl

5 http://www.nitrc.org/projects/conn

6 https://jasp-stats.org/
