# Supplementary material for: Sex Effect on Presurgical Language Mapping in Patients With a Brain Tumor
Source: Front Neurosci. 2020 Jan 24;14:4. doi: 10.3389/fnins.2020.00004 (PMC6992642; doi:10.3389/fnins.2020.00004)
Supplement: Supplementary file 1 [file Data_Sheet_1.PDF]

Supplemental Materials

S1. Information on MRI scanners used in the study.

Scanner	Type	Number of patients	Parameters
Scanner 1	Siemens Trio	18	TR/TE = 2000/30 ms, flip angle = 85°<br>FOV= 220 mm × 220 mm<br>Matrix = 64 × 64, 24 slices<br>Voxel size = 3.44 × 3.44 × 5.0 mm^3<br>Ascending interleaved sequence
Scanner 2	Siemens Verio	9	TR/TE = 2000/30 ms, flip angle = 85°<br>FOV= 220 mm × 220 mm<br>Matrix = 64 × 64, 24 slices<br>Voxel size = 3.44 × 3.44 × 5.0 mm^3<br>Ascending interleaved sequence
Scanner 3	Siemens Skyra	12	TR/TE = 2000/30 ms, flip angle = 85°<br>FOV= 220 mm × 220 mm<br>Matrix = 64 × 64, 32 slices<br>Voxel size = 3.44 × 3.44 × 4.0 mm^3<br>Ascending interleaved sequence
Scanner 4	Siemens Prisma	8	TR/TE = 2000/30 ms, flip angle = 85°<br>FOV= 220 mm × 220 mm<br>Matrix = 64 × 64, 32 slices<br>Voxel size = 3.44 × 3.44 × 4.0 mm^3<br>Ascending interleaved sequence

S2. An example of the optimized spatial normalization procedure in a patient with a left frontal glioblastoma.

A conservative brain tumor mask that included a prior resection cavity (determined by its high density and proximity to the tumor boundary) was defined in the gadolinium-enhanced T1 images. The brain tissue was extracted using the Optimized Brain Extraction method (optiBET). The extracted brain images were then normalized to the Montreal Neurological Institute MNI-152 template. The resulting transformation matrix was used to normalize the functional images that were coregistered to the T1 images.

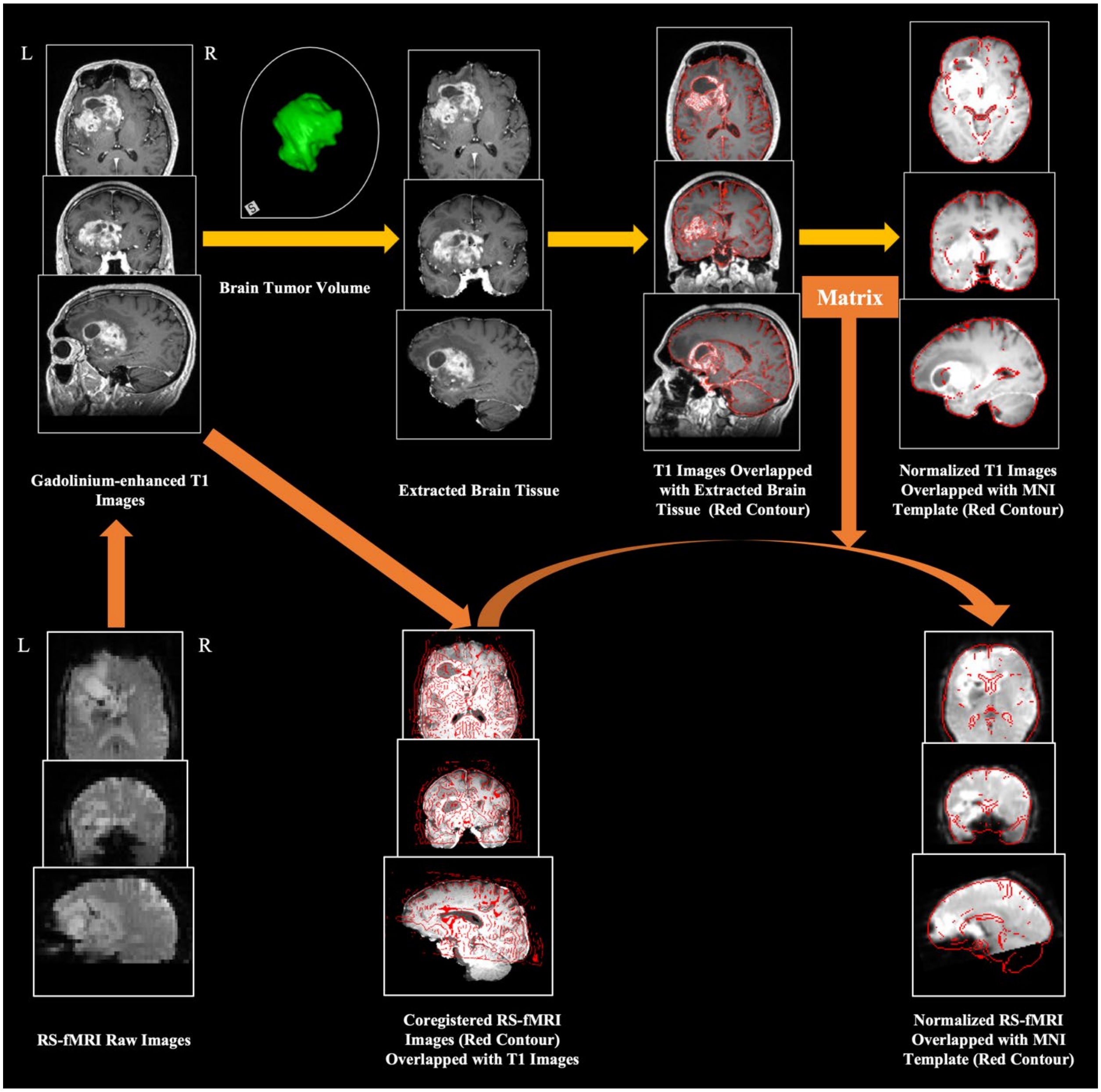

S3. Main effect of language task across sex.

Cluster Size	Brain Regions	L/R	BA	Peak MNI Coordinates			Z-values
				x	y	z	
**SC > AG**							
5303	Angular gyrus	R	39	50	-66	34	13.73
	Precuneus	L	31	-4	-50	36	10.76
1469	Middle frontal gyrus	L	8	-24	30	48	7.23
1211	Angular gyrus	L	39	-44	-72	32	11.08
273	Middle frontal gyrus	R	8	30	30	50	8.69
**AG > SC**							
1282	Cuneus	R	19	14	-90	24	11.06
739	Postcentral gyrus	L	4	-58	-8	22	5.15
519	Inferior parietal lobule	R	7	34	-46	42	5.56
374	Precentral gyrus	R	6	56	0	52	7.97
335	Inferior frontal gyrus, pars opercularis	R	44	44	10	24	6.14
221	Middle frontal gyrus	L	6	-2	10	50	12.66

Z-score> 3.1, cluster size > 200 voxels, corrected p < 0.05.

BA, Brodmann area; MNI, Montreal Neurological Institute; SMA, supplemental motor area; L, left; R, right.

The areas rendered by the red-yellow color scale indicate greater activation during the sentence completion (SC) task, and the areas rendered by the blue-light blue color scale indicate greater activation during the AG task (Z-score> 3.1, corrected P < 0.05).

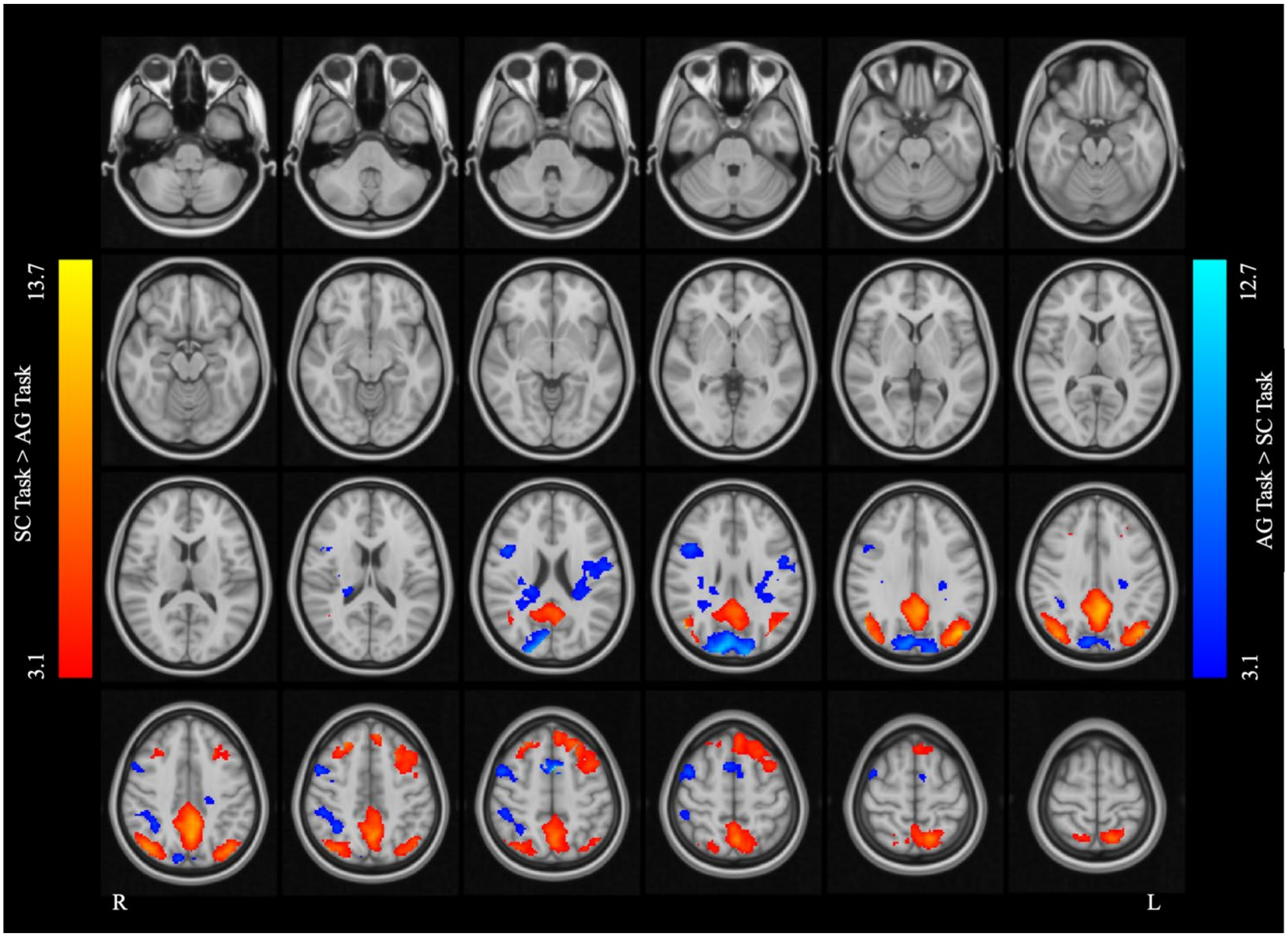

S4. The interaction of sex and language task.

Cluster Size	Brain Regions	L/R	BA	Peak MNI Coordinates			Z-values
				x	y	z	
1081	Precuneus	R	7	10	-48	38	5.42
438	Angular gyrus	R	39	42	-72	46	5.23
251	Superior occipital gyrus	R	19	24	-82	44	4.91
203	SMA	L	6	-4	22	56	4.31

Z-score> 3.1, cluster size > 200 voxels, corrected p < 0.05.

BA, Brodmann area; MNI, Montreal Neurological Institute; SMA, supplemental motor area; L, left; R, right.

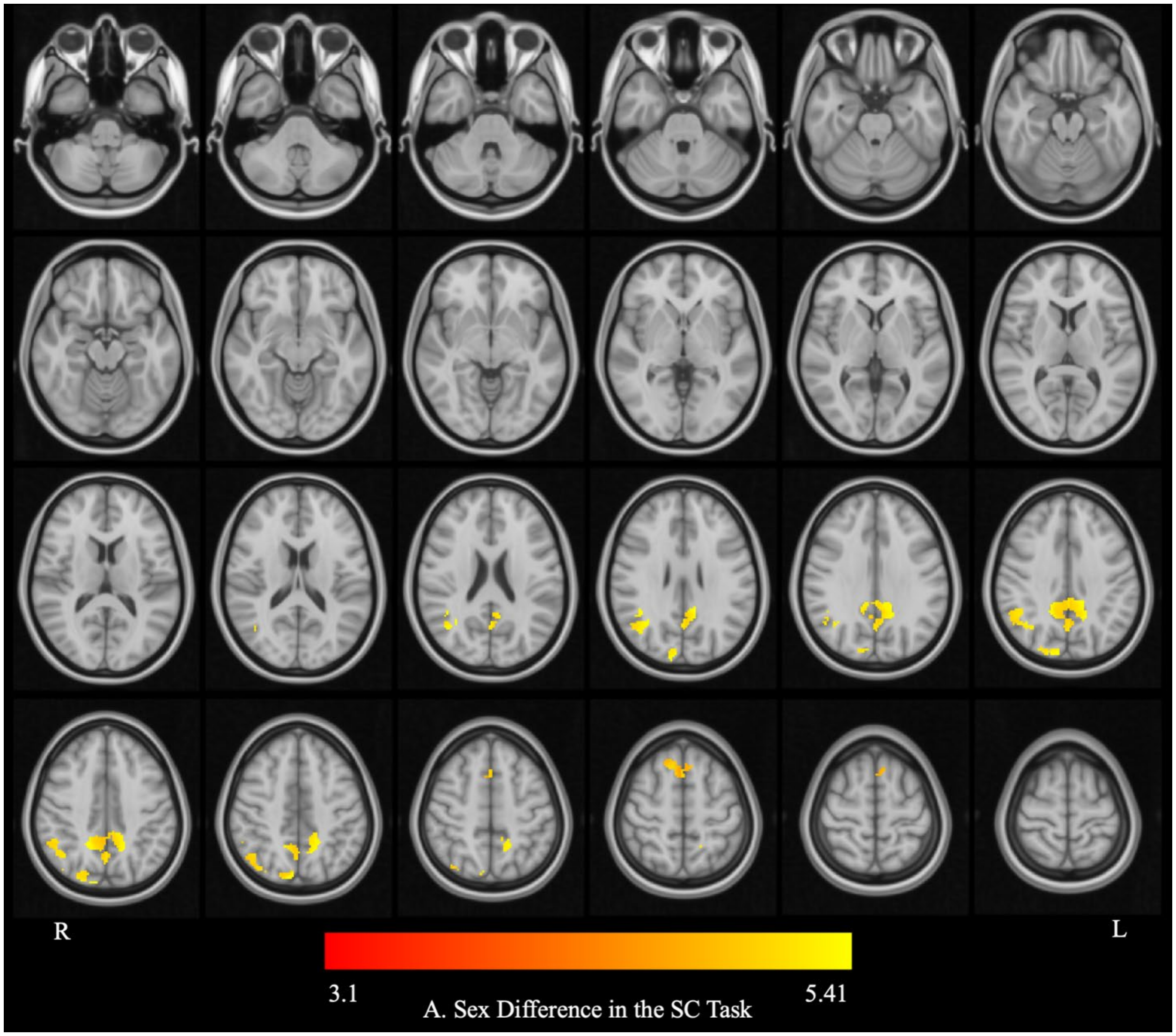

A. Sex Difference in the SC Task
